# Optimal delivery time for patients with diet-controlled gestational diabetes mellitus: a single-center real-world study

**DOI:** 10.1186/s12884-022-04683-2

**Published:** 2022-04-23

**Authors:** Zongzhi Yin, Tengteng Li, Lu Zhou, Jiajia Fei, Jingjing Su, Dan Li

**Affiliations:** 1 Department of Obstetrics and Gynecology, The First Affiliated Hospital of Anhui Medical University, Hefei, 230022 China; 2 Department of Obstetrics and Gynecology, Chaohu Hospital of Anhui Medical University, Chaohu, 238001 China; 3 NHC Key Laboratory of the Study of Abnormal Gametes and the Reproductive Tract, Anhui Medical University, Hefei, 230022 China; 4 Department of Scientific Research, The Second Affiliated Hospital of Anhui Medical University, Hefei, 230601 China

**Keywords:** Gestational diabetes mellitus, Optimal delivery time, Maternal–fetal outcome, Iatrogenic intervention, Uterine contraction

## Abstract

**Background:**

To determine the optimal delivery time for women with diet-controlled gestational diabetes mellitus by comparing differences in adverse maternal–fetal outcome and cesarean section rates.

**Methods:**

This real-world retrospective study included 1,050 patients with diet-controlled gestational diabetes mellitus who delivered at 35–42 weeks’ gestation. Data on patient characteristics, maternal–fetal outcomes, and cesarean section rate based on fetal gestational age were collected and analyzed. Differences between deliveries with and without iatrogenic intervention were also analyzed.

**Results:**

The cesarean section rate at ≥ 41 weeks’ gestation was significantly higher than that at 39–39 + 6 weeks (56% vs. 39%, *p* = 0.031). There were no significant differences in multiple adverse maternal or neonatal outcomes at delivery before and after 39 weeks. Vaginal delivery rates were increased significantly at 39–39 + 6 weeks due to iatrogenic intervention (*p* = 0.005) and 40–40 + 6 weeks (*p* = 0.003) in patients without and with spontaneous uterine contractions, respectively.

**Conclusions:**

It’s recommended that optimal delivery time for patients with diet-controlled gestational diabetes mellitus should be between 39- and 40 + 6 weeks’ gestation. Patients who have Bishop scores higher than 4 can undergo iatrogenic intervention at 39–39 + 6 weeks. However iatrogenic interventions are not recommended for patients with low Bishop scores.

## Background

Gestational diabetes mellitus (GDM) is defined as the onset or first recognition of glucose intolerance during pregnancy [1]. This condition is one of the most common obstetrical complications of pregnancy; with its prevalence varying substantially worldwide from 1.8% to more than 31.0%, which continues to increase [2, 3]. The blood glucose levels in women with GDM can usually be controlled through diet, exercise, or medication. Between 70–85% of women achieve euglycemia through diet or exercise and this is referred to as diet-controlled GDM. Patients who control hyperglycemia using medication are regarded as having medication-treated GDM. The number of patients with diet-controlled GDM is much higher than that of patients with medication-treated GDM [1, 2, 4].

Previous studies [1, 2, 5–7] have shown that the incidence of maternal and fetal complications significantly increases in cases involving GDM. Maternal and fetal complications include premature rupture of membrane (PROM) (22.6% vs. 11.5%), perineal laceration (18% vs. 10.7%), hypoglycemia (4.3% vs. 0.03%), shoulder dystocia/birth injury (4.3% vs. 2.6%), macrosomia (20.63% vs. 4.0%), and neonatal respiratory distress (14.55% vs. 3.0%), respectively. Furthermore, in several studies [8, 9], the incidence of complications was higher in patients with medication-treated GDM than in those with diet-controlled GDM (i.e., a 58% vs. 40% cesarean section rate).

Glycemic management is the main measure to reduce the occurrence of GDM complications in pregnant women. The optimal time and mode of delivery for patients with GDM is primarily based on glycemic control. Recommendations for women with medication-treated GDM are consistent across multiple clinical guidelines calling to proceed for the induction of labor at 39–39 + 6 weeks of gestation [2, 10]. However, various guidelines for diet-controlled GDM do not provide clear recommendations for optimal delivery time [11]. The 2018 American College of Obstetricians and Gynecologists (ACOG) guidelines recommend that delivery in women with diet-controlled GDM should not be induced before 39 weeks of gestation, which leaves a large span of time from 39 to 41 weeks for delivery. The guidelines of the 2020 Society of Obstetricians and Gynecologists of Canada (SOGC) suggest that induction by the 40-week mark may be beneficial for this population [2, 10]. Furthermore, the studies on which the guideline recommendations are based also have limitations. The 2018 ACOG guideline is based on the outcome of a multicenter open-label randomized controlled trial that was published in the British Journal of Obstetrics and Gynecology; it included only 425 patients with an overrepresentation of white individuals (73.6%), which may have produced false-negative results that might have been a limitation [12]. The 2020 SOGC guidelines are based on the results of a retrospective cohort study [13] which was published in the International Journal of Gynecology and Obstetrics. In this study, the differences in stillbirth rates between women with and without diet-controlled GDM were compared. No difference between groups was found before 40 weeks of gestation, but there was a significantly lower stillbirth rate after 40 weeks in those with diet-controlled GDM. However, that study illustrated that women with diet-controlled GDM were routinely induced at 40 weeks of gestation. It was not clearly stated whether there were differences in the clinical characteristics of women who delivered after 40 weeks of gestation. It was also not indicated whether iatrogenic intervention should be implemented before 41 weeks of gestation according to GDM status or if there was an optimal delivery time between 39 and 41 weeks of gestation. Hence, this study aimed to determine the optimal delivery time for women with diet-controlled diabetes mellitus by comparing differences in adverse maternal–fetal outcome and cesarean section rates.

## Methods

### Study Population

This retrospective cohort study included 1,050 singleton pregnancies with diet-controlled GDM. All patients were hospitalized in the Department of Obstetrics and Gynecology of The First Affiliated Hospital of Anhui Medical University between January 1, 2018, and December 31, 2020 and all included patients were of Asian origin. The study design was approved by the First Affiliated Hospital of Anhui Medical University Ethics Committee for the Protection of Human Subjects in Research (PJ2020-06–12). The need for acquisition of informed consent was waived owing to the retrospective nature of this study. All methods were carried out in accordance with relevant guidelines and regulations.

Diagnosis of patients with GDM was made according to the Chinese Society of Obstetrics and Gynecology and the Chinese Medical Association consensus [14]. One or more of the following values from a 75-g oral glucose tolerance test (OGTT) between the 24th and 28th week of gestation must be measured to meet the diagnostic criteria for GDM: fasting plasma glucose (FPG) ≥ 5.1 mmol/L (92 mg/dL), 1-h plasma glucose ≥ 10.0 mmol/L (180 mg/dL), and 2-h plasma glucose ≥ 8.5 mmol/L (153 mg/dL). Patients with GDM who followed standard management of their diet and exercise performed blood glucose control for 2 weeks after which their plasma glucose levels were assessed again. Patients who maintained a fasting glucose of ≤ 5.3 mmol/L and a 2-h postprandial glucose of ≤ 6.7 mmol/L were considered well-controlled and were included in our study as patients with diet-controlled GDM. These patients underwent standard diet management and weekly plasma glucose checks until delivery. The treatment was adjusted according to the conditions and needs of the woman.

Patients aged 18 years or older with a singleton pregnancy, vertex presentation, and who were diagnosed with GDM with well-controlled blood glucose in their current pregnancy were included. Patients with any of the following conditions were excluded from the study: gestational age at birth of less than 35 weeks’ gestation, pregestational diabetes mellitus, GDM patients requiring medication control, multiple pregnancies, placenta previa, scarred uterus, fetal malposition (transverse, breech), and medical/surgical comorbidity as an indication for cesarean section. All gestational ages were verified using the last menstrual period and confirmed using the first trimester sonographic measurement of crown-rump length.

All patients delivered at ≥ 35 weeks of gestation, and the patient groups were formed according to the week of gestation at delivery. Patients who delivered at 35–36 + 6 weeks comprised the < 37 gestational week group. The patient clinical information and adverse perinatal maternal and neonatal outcomes were collected at < 37 W, 37–37 + 6 W, 38–38 + 6 W, 39–39 + 6 W, 40–40 + 6 W, and ≥ 41 weeks’ gestation.

### Outcome Measures

We divided all outcomes into two categories: pregnancy complications and maternal outcomes, and fetal and neonatal outcomes. All diagnostic criteria followed the clinical guidelines published by the Chinese Society of Obstetrics and Gynecology and the Chinese Medical Association. All the diagnoses were made by a senior obstetrician to reduce the error and ensure data accuracy.

### Pregnancy Complications and Maternal Outcomes

A preeclampsia diagnosis was made according to the “Diagnosis and management of hypertension in pregnancy (2020) [15],” which was defined by at least two outcomes: > 4-h interval with systolic blood pressure ≥ 140 mmHg and/or diastolic blood pressure ≥ 90 mmHg and random positive urine protein of ≥ 0.3 g/24 h. We defined PROM according to the “Diagnosis and Management of Premature Rupture of Membranes (2015) [16]”—which defines a rupture of membranes before the onset of labor and vaginal fluid flow as premature rupture after 28 weeks of gestation. The definition of postpartum hemorrhage (PPH) was based on the “Prevention and Treatment of Postpartum Hemorrhage (2014) [17],” which states that a PPH is the blood loss of more than 500 mL within 24 h after delivery of the fetus in vaginal delivery and more than 1000 mL in cesarean delivery. An amniotic fluid volume of less than 300 mL, a maximum vertical depth of amniotic fluid ≤ 2 cm, and an amniotic fluid index (AFI) ≤ 5 cm in late pregnancy was considered as oligohydramnios. In cases of yellowish-green or brownish green amniotic fluid, with a large amount of meconium or paste-like sticky meconium, III-polluted amniotic fluid was considered.

Perineal laceration was defined as any injury in the genital area that occurred from lacerations during labor, involving the skin of the perineum and vaginal mucosa to the deeper rectal mucosa. Poor healing of the perineum was defined as the manifestation of fever, painful incision, yellow purulent secretions, and positive bacterial culture within 3 days of delivery. Obstructed shoulder delivery was diagnosed when there was a failure to deliver the fetal shoulder(s) with a gentle downward traction on the fetal head, resulting in the requirement of additional obstetric maneuvers for delivery of the fetus. Postoperative pyrexia was defined as two recordings of an oral temperature higher than 38 °C daily (at 4-h intervals) measured within 10 days after delivery.

### Fetal and Neonatal Outcomes

We defined asphyxia neonatorum according to the “Expert Consensus on the Diagnosis of Neonatal Asphyxia (2016) [18],” which is defined as a 1-min Apgar score ≤ 7- or 5-min Apgar score ≤ 7 with a cord arterial blood pH < 7.2. The definition of neonatal respiratory distress was in accordance with that in the “Diagnosis and Treatment of Neonatal Respiratory Distress (2013) [19],” which includes the following criteria: 1) acute onset in neonates; 2) clear perinatal triggers; 3) typical clinical signs (progressive dyspnea, cyanosis, and reduced or absent lung breath sounds); and 4) an arterial blood gas analysis of Pa(O_2_)/FiO_2_ ≤ 200 mmHg. The diagnosis of fetal distress was made according to the following criteria: 1) abnormal fetal heart rate (fetal heart rate 100 bpm), 2) baseline variability ≤ 5 bpm, 3) fetal movements < 10 times/12 h or absent fetal movements. A fetal birth weight ≥ 4000 g was considered fetal macrosomia. If the fetal serum glucose level was lower than 2.6 mmol/L within 24 to 72 h after delivery neonatal hypoglycemia was considered.

### Decision of Delivery

The decision for timing and mode of delivery was determined based on two of the following scenarios: 1) for patients who were admitted to the hospital with regular uterine contraction and treated according to the normal course of labor; and 2) for those who had pregnancy complications, final decisions were made by a senior obstetrician based on the clinical guidelines for the management of the related diseases. The indication for iatrogenic intervention was based on the patient's Bishop score and thegestational age. The Chinese GDM bulletin was followed. Iatrogenic interventions were given before 41 weeks of gestation for these patients without spontaneous uteri contractions and the Bishop scores were evaluated [14]. If the score exceeded 4, low-dose oxytocin or dinoprostone was used to induce labor. For patients with a low Bishop score, balloon was used for cervical dilation.

### Statistical Analysis

SPSS version 19.0 was used for the statistical processing of the data. The measurement data are expressed as the mean ± standard deviation, and the counting data as numbers (percentages). Mean values were compared between multiple groups of measurement data. One-way analysis of variance was used to conform data to a normal distribution, and Student–Newman–Keuls tests were used for pairwise comparisons among groups. Data that were not normally distributed were analyzed using rank-sum tests. Two groups were analyzed using the t-test. Count data were analyzed using the χ^2^ test, and statistical significance was set at *p* < 0.05. Spearman and Pearson methods were used for the correlation analyses.

## Results

### Patient Characteristics

Table 1 shows patient characteristics according to weeks of gestation at delivery. Maternal age in the 37–37 + 6 gestational weeks group was significantly higher than that in the 39–39 + 6 group (*p* = 0.005), while maternal age in the 40–40 + 6 and ≥ 41 groups was significantly lower than that in the 39–39 + 6 group (*p* = 0.001 and 0.036, respectively). Maternal body mass indices (BMIs) before pregnancy in the 37–37 + 6 and ≥ 41 groups were significantly higher than in the 39–39 + 6 group (*p* = 0.022 and 0.002, respectively). The changes in BMI during pregnancy were not significantly different between groups.

**Table 1:** Patient Characteristics

Gestational Week	39–39^+6^ W	< 37 W	< 37 W	37–37^+6^ W	38–38^+6^ W	40–40^+6^ W	≥ 41 W
N	372	40	40	74	220	294	50
Age (Years)							
Mean ± SD	29.5 ± 3.41	28.8 ± 3.91	28.8 ± 3.91	30.8 ± 4.54	29.4 ± 3.43	28.7 ± 3.08	28.5 ± 3.23
Median	29.0	29.0	29.0	30.0	29.0	29.0	28.0
(Min, Max)	(19, 43)	(21, 35)	(21, 35)	(23, 48)	(20, 39)	(16, 39)	(21, 38)
P-value		0.188	0.188	0.005	0.599	0.001	0.036
BMI before Pregnant (kg/m^2^)							
Mean ± SD	22.01 ± 3.418	22.95 ± 3.782	22.95 ± 3.782	23.05 ± 3.674	22.34 ± 3.459	22.16 ± 3.164	23.73 ± 4.019
Median	21.60	22.40	22.40	23.05	21.90	21.80	23.85
(Min, Max)	(15.1, 47.8)	(14.1, 34.8)	(14.1, 34.8)	(16.0, 39.3)	(14.0, 37.3)	(12.5, 34.2)	(17.4, 37.7)
P-value		0.122	0.122	0.022	0.265	0.586	0.002
BMI change during Pregnant (kg/m^2^)							
Mean ± SD	5.45 ± 1.719	5.06 ± 1.744	5.06 ± 1.744	5.02 ± 1.615	5.42 ± 1.853	5.60 ± 1.832	5.47 ± 1.533
Median	5.40	5.00	5.00	4.80	5.40	5.50	5.55
(Min, Max)	(0.0, 11.6)	(0.7, 9.4)	(0.7, 9.4)	(1.6, 8.4)	(0.0, 12.5)	(0.0, 12.0)	(1.9, 8.7)
P-value		0.197	0.197	0.055	0.816	0.289	0.944

BMI: Body mass index; SD: Standard deviation

### Maternal Outcomes

Table 2 shows maternal delivery outcomes (by weeks of gestation at delivery) in patients with diet-controlled GDM; we found that the rates of PROM at < 37 and 37–37 + 6 gestational weeks were significantly higher and at 40–40 + 6 weeks were significantly lower than those at 39–39 + 6 weeks (*p* < 0.001, 0.036, and 0.020, respectively). The incidence of cesarean delivery at ≥ 41 gestational weeks was significantly higher than that at 39–39 + 6 weeks (56% vs. 39%, *p* = 0.031), but the rate of perineal laceration was significantly lower (*p* = 0.005). The incidences of GDM -related complications, poor perineal healing, obstructed shoulder delivery, PPH, and postoperative fever were not significantly different between the groups.

**Table 2:** Maternal Outcomes

Gestational Week	39–39^+6^ W	< 37 W	37–37^+6^ W	38–38^+6^ W	40–40^+6^ W	≥ 41 W
PROM (N)	372	40	74	220	294	50
Yes(n, %)	90 (24.2%)	23 (57.5%)	28 (37.8%)	67 (30.5%)	51 (17.3%)	7 (14.0%)
No(n, %)	282 (75.8%)	17 (42.5%)	46 (62.2%)	153 (69.5%)	243 (82.7%)	43 (86.0%)
Risk Diff (95% CI)		33.31% (17.38%,49.2%)	13.64% (1.77%,25.52%)	6.26% (-1.22%,13.74%)	-6.85%(-12.98%,-0.71%)	-10.19% (-20.75%,0.36%)
P-value		0.000	0.020	0.102	0.036	0.151
Delivery Method (N)	372	40	74	220	294	50
C-section(n, %)	145 (39.0%)	16 (40.0%)	33 (44.6%)	104 (47.3%)	127 (43.2%)	28 (56.0%)
Natural Delivery(n, %)	227 (61.0%)	24 (60.0%)	41 (55.4%)	116 (52.7%)	167 (56.8%)	22 (44.0%)
Risk Diff (95% CI)		1.02% (-14.95%,16.9%)	5.62% (-6.75%,17.98%)	8.29% (0.04%, 16.55%)	4.22% (-3.31%,11.74%)	17.02% (2.40%,31.65)
P-value		1.000	0.367	0.058	0.302	0.031
GDM-related Complications (N)	372	40	74	220	294	50
Yes(n, %)	70 (18.8%)	9 (22.5%)	10 (13.5%)	37 (16.8%)	56 (19.0%)	13 (26.0%)
No(n, %)	302 (81.2%)	31 (77.5%)	64 (86.5%)	183 (83.2%)	238 (81.0%)	37 (74.0%)
Risk Diff (95% CI)		3.68% (-9.85%,17.22%)	-5.30% (-14.05%,3.44%)	-2.00% (-8.34%, 4.34%)	0.23% (-5.76%, 6.22%)	7.18% (-5.61%,19.97%)
P-value		0.532	0.322	0.582	1.000	0.255
Perineal laceration (N)	227	24	41	116	167	22
Yes(n, %)	147 (64.8%)	18 (75.0%)	29 (70.7%)	79 (68.1%)	94 (56.3%)	7 (31.8%)
No(n, %)	80 (35.2%)	6 (25.0%)	12 (29.3%)	37 (31.9%)	73 (43.7%)	15 (68.2%)
Risk Diff (95% CI)		10.24% (-8.16%,28.65%)	5.97% (-9.28%,21.22%)	3.35% (-7.17%,13.86%)	-8.47% (-18.23%,1.29%)	-32.94%(-53.37%,12.5%)
P-value		0.372	0.592	0.550	0.095	0.005
Poor healing of perineal (N)	227	24	41	116	167	22
Yes(n, %)	1 (0.4%)	0	1 (2.4%)	1 (0.9%)	2 (1.2%)	1 (4.5%)
No(n, %)	226 (99.6%)	24 (100.0%)	40 (97.6%)	115 (99.1%)	165 (98.8%)	21 (95.5%)
Risk Diff (95% CI)		0.44% (-0.42%, 1.30%)	2.00% (-2.80%, 6.80%)	0.42% (-1.47%, 2.31%)	0.76% (-1.10%, 2.62%)	4.10% (-4.64%,12.85%)
P-value		1.000	0.283	1.000	0.577	0.169
Shoulder dystocia (N)	227	24	41	116	167	22
Yes(n, %)	7 (3.1%)	0	0	3 (2.6%)	2 (1.2%)	0
No(n, %)	220 (96.9%)	24 (100.0%)	41 (100.0%)	113 (97.4%)	165 (98.8%)	22 (100.0%)
Risk Diff (95% CI)		3.08% (0.83%, 5.33%)	3.08% (0.83%, 5.33%)	-0.50% (-4.16%, 3.16%)	-1.89% (-4.68%, 0.90%)	3.08% (0.83%, 5.33%)
P-value		1.000	0.600	1.000	0.312	1.000
Postoperative pyrexia (N)	145	16	33	104	127	28
Yes(n, %)	3 (2.1%)	1 (6.3%)	1 (3.0%)	1 (1.0%)	3 (2.4%)	1 (3.6%)
No(n, %)	142 (97.9%)	15 (93.8%)	32 (97.0%)	103 (99.0%)	124 (97.6%)	27 (96.4%)
Risk Diff (95% CI)		4.20% (-7.89%, 16.28%)	0.98% (-5.31%, 7.26%)	-1.09% (-4.06%, 1.88%)	0.31% (-3.20%, 3.81%)	1.52% (-5.73%, 8.77%)
P-value		0.343	0.561	0.643	1.000	0.508
Postpartum Hemorrhage (N)	372	40	74	219	294	50
Yes(n, %)	21 (5.6%)	0	5 (6.8%)	15 (6.8%)	21 (7.1%)	3 (6.0%)
No(n, %)	351 (94.4%)	40 (100.0%)	69 (93.2%)	204 (93.2%)	273 (92.9%)	47 (94.0%)
Risk Diff (95% CI)		5.65% (3.30%, 7.99%)	1.11% (-5.07%, 7.29%)	1.20% (-2.88%, 5.29%)	1.50% (-2.27%, 5.26%)	0.35% (-6.63%, 7.34%)
P-value		0.246	0.785	0.595	0.428	1.000

PROM: Premature rupture of membrane; GDM: Gestational diabetes mellitus; CI: Confidence interval

### Neonatal Outcomes

Table 3 shows neonatal outcomes (by weeks of gestation at delivery) in patients with diet-controlled GDM. The results showed that the rate of macrosomia was not significantly different between the 39–39 + 6 gestational weeks group and other groups, except that no macrosomia was present at < 37 weeks. Compared with the 39–39 + 6 gestational weeks group, the rate of III-polluted amniotic fluid rate at 37–37 + 6 weeks was significantly lower (*p* = 0.033). The incidences of postnatal hypoglycemia and vomiting and moaning at < 37 gestational weeks were significantly higher than those in the 39–39 + 6 gestational weeks group (*p *= 0.049 and 0.028, respectively); these incidences were not significantly different from those in the other gestational week groups. There were no significant differences in the incidence of neonatal asphyxia, neonatal respiratory distress syndrome, or neonatal malformations.

**Table 3:** Neonatal Outcomes

Gestational Week	39–39^+6^ W	< 37 W	37–37^+6^ W	38–38^+6^ W	40–40^+6^ W	≥ 41 W
Weight N	40	74	220	372	293	50
Mean ± SD(g)	2766 ± 348.86	3154 ± 444.46	3317 ± 430.14	3416 ± 424.87	3550 ± 379.32	3630 ± 440.42
Median	2750	3100	3250	3400	3500	3575
(Min, Max)	(2200, 3950)	(2110, 4300)	(2250, 5200)	(2250, 4800)	(2500, 4700)	(2700, 4700)
Fetal macrosomia (N)	369	40	74	219	294	50
Yes(n, %)	38 (10.3%)	0	4 (5.4%)	15 (6.8%)	38 (12.9%)	10 (20.0%)
No(n, %)	331 (89.7%)	40 (100.0%)	70 (94.6%)	204 (93.2%)	256 (87.1%)	40 (80.0%)
Risk Diff (95% CI)		10.30% (7.20%,13.40%)	-4.89% (-10.91%,1.12%)	-3.45% (-8.01%, 1.11%)	2.63% (-2.30%, 7.56%)	9.70% (-1.81%,21.21%)
P-value		0.038	0.275	0.181	0.327	0.056
III-polluted amniotic fluid (N)	372	40	74	220	294	50
Yes(n, %)	21 (5.6%)	0	0	5 (2.3%)	24 (8.2%)	4 (8.0%)
No(n, %)	351 (94.4%)	40 (100.0%)	74 (100.0%)	215 (97.7%)	270 (91.8%)	46 (92.0%)
Risk Diff (95% CI)		5.65% (3.30%, 7.99%)	5.65% (3.30%, 7.99%)	-3.37% (-6.43%, -0.31%)	2.52% (-1.39%, 6.43%)	2.35% (-5.52%,10.23%)
P-value		0.246	0.033	0.062	0.216	0.520
Hypoglycemia (N)	372	40	74	220	294	50
Yes(n, %)	2 (0.5%)	2 (5.0%)	1 (1.4%)	5 (2.3%)	2 (0.7%)	1 (2.0%)
No(n, %)	370 (99.5%)	38 (95.0%)	73 (98.6%)	215 (97.7%)	292 (99.3%)	49 (98.0%)
Risk Diff (95% CI)		4.46% (-2.33%,11.26%)	0.81% (-1.92%, 3.55%)	1.74% (-0.37%, 3.84%)	0.14% (-1.06%, 1.34%)	1.46% (-2.49%, 5.41%)
P-value		0.049	0.421	0.108	1.000	0.316
Spitting and groaning (N)	372	40	74	220	294	50
Yes(n, %)	14 (3.8%)	5 (12.5%)	2 (2.7%)	3 (1.4%)	10 (3.4%)	0
No(n, %)	358 (96.2%)	35 (87.5%)	72 (97.3%)	217 (98.6%)	284 (96.6%)	50 (100.0%)
Risk Diff (95% CI)		8.74% (-1.69%,19.17%)	-1.06% (-5.23%, 3.11%)	-2.40% (-4.87%, 0.07%)	-0.36% (-3.20%, 2.47%)	3.76% (1.83%,5.70%)
P-value		0.028	1.000	0.126	0.838	0.390
Asphyxia Neonatorum (N)	372	40	74	220	294	50
Yes(n, %)	3 (0.8%)	0	1 (1.4%)	2 (0.9%)	4 (1.4%)	1 (2.0%)
No(n, %)	369 (99.2%)	40 (100.0%)	73 (98.6%)	218 (99.1%)	290 (98.6%)	49 (98.0%)
Risk Diff (95% CI)		0.81% (-0.10%,1.72%)	0.54% (-2.24%, 3.33%)	0.10% (-1.45%, 1.65%)	0.55% (-1.05%, 2.16%)	1.19% (-2.79%, 5.18%)
P-value		1.000	0.517	1.000	0.705	0.397
Neonatal Respiratory Distress (N)	372	40	74	220	294	50
Yes(n, %)	0	0	0	0	1 (0.3%)	0
No(n, %)	372 (100.0%)	40 (100.0%)	74 (100.0%)	220(100.0%)	293 (99.7%)	50(100.0%)
Risk Diff (95% CI)		0	0	0	0.34% (-0.33%, 1.01%)	0
P-value		1.000	1.000	1.000	0.441	1.000
Neonatal Malformation (N)	372	40	74	220	294	50
Yes(n, %)	3 (0.8%)	0	0	0	2 (0.7%)	0
No(n, %)	369 (99.2%)	40 (100.0%)	74 (100.0%)	220(100.0%)	292 (99.3%)	50(100.0%)
Risk Diff (95% CI)		0.81% (-0.10%,1.72%)	0.81% (-0.10%, 1.72%)	0.81% (-0.10%, 1.72%)	-0.13% (-1.43%, 1.18%)	0.81% (-0.10%, 1.72%)
P-value		1.000	1.000	1.000	0.298	1.000

SD: Standard deviation; CI: Confidence interval

### Indications for Cesarean Section

As shown in Table 4, compared with the 39–39 + 6 gestational weeks group, the rate of cesarean delivery due to macrosomia at < 37 and 38–38 + 6 weeks was significantly lower (*p* = 0.040 and 0.022, respectively). The indication due to oligohydramnios at 37–37 + 6, 38–38 + 6, and ≥ 41 weeks was significantly higher (*p* = 0.034, 0.020, and 0.025, respectively), and the indication due to preeclampsia at < 37 weeks was significantly higher than that in the 39–-39 + 6 gestational weeks group (*p* = 0.015).

**Table 4:** Delivery Information

Gestational Week	39–39^+6^ W	< 37 W	37–37^+6^ W	38–38^+6^ W	40–40^+6^ W	≥ 41 W
*Indications for Cesarean Section* Macrosomia (N)	46	8	18	44	36	7
Yes(n, %)	19 (41.3%)	0	3 (16.7%)	8 (18.2%)	19 (52.8%)	2 (28.6%)
No(n, %)	27 (58.7%)	8 (100.0%)	15 (83.3%)	36 (81.8%)	17 (47.2%)	5 (71.4%)
Risk Diff (95% CI)		41.30% (27.08%,55.53%)	-24.64% (-46.97%, -2.30%)	-23.12%(-41.35%, -4.89%)	11.47% (-10.17%,33.12%)	-12.73% (-49.10%,23.63%)
P-value		0.040	0.082	0.022	0.374	0.690
Fetal distress (N)	46	8	18	44	36	7
Yes(n, %)	9 (19.6%)	2 (25.0%)	1 (5.6%)	5 (11.4%)	6 (16.7%)	0
No(n, %)	37 (80.4%)	6 (75.0%)	17 (94.4%)	39 (88.6%)	30 (83.3%)	7 (100.0%)
Risk Diff (95% CI)		5.43% (-26.69%,37.5%)	-14.01%(-29.61%,1.59%)	-8.20% (-23.01%,6.61%)	-2.90% (-19.62%,13.8%)	19.57% (8.10%,31.03%)
P-value		0.659	0.259	0.386	0.781	0.334
GDM (N)	46	8	18	44	36	7
Yes(n, %)	7 (15.2%)	1 (12.5%)	3 (16.7%)	13 (29.5%)	2 (5.6%)	1 (14.3%)
No(n, %)	39 (84.8%)	7 (87.5%)	15 (83.3%)	31 (70.5%)	34 (94.4%)	6 (85.7%)
Risk Diff (95% CI)		-2.72% (-27.88%,22.4%)	1.45% (-18.65%,21.5%)	14.33% (-2.69%,31.34%)	-9.66% (-22.46%,3.13%)	-0.93% (-28.86%,26.9%)
P-value		1.000	1.000	0.131	0.286	1.000
Oligohydramnios (N)	46	8	18	44	36	7
Yes(n, %)	3 (6.5%)	0	5 (27.8%)	11 (25.0%)	8 (22.2%)	3 (42.9%)
No(n, %)	43 (93.5%)	8 (100.0%)	13 (72.2%)	33 (75.0%)	28 (77.8%)	4 (57.1%)
Risk Diff (95% CI)		6.52% (-0.61%,13.66%)	21.26% (-0.63%,43.14%)	18.48% (3.83%,33.13%)	15.70% (0.36%,31.04%)	36.34% (-1.01%,73.68%)
P-value		1.000	0.034	0.020	0.052	0.025
Preeclampsia (N)	46	8	18	44	36	7
Yes(n, %)	8 (17.4%)	5 (62.5%)	6 (33.3%)	7 (15.9%)	3 (8.3%)	1 (14.3%)
No(n, %)	38 (82.6%)	3 (37.5%)	12 (66.7%)	37 (84.1%)	33 (91.7%)	6 (85.7%)
Risk Diff (95% CI)		45.11% (9.82%,80.40%)	15.94% (-8.43%,40.32%)	-1.48% (-16.87%,13.9%)	-9.06% (-23.25%,5.14%)	-3.11% (-31.25%,25.0%)
P-value		0.015	0.190	1.000	0.332	1.000
*Trial of labor* N	256	25	44	122	203	29
Vaginal Delivery (n, %)	227 (88.7%)	24(96.0%)	41 (93.2%)	116 (95.1%)	167 (82.3%)	22 (75.9%)
Natural Labor switched to C-section (n, %)	29 (11.3%)	1 (4.0%)	3 (6.8%)	6 (4.9%)	36 (17.7%)	7 (24.1%)
Risk Diff (95% CI)		-7.33% (-15.93%,1.28%)	-4.51% (-12.91%,3.89%)	-6.41% (-11.87%,0.95%)	6.41% (-0.13%,12.94%)	12.81% (-3.24%,28.86%)
P-value		0.493	0.596	0.056	0.059	0.071
*Natural Labor Rate*						
Iatrogenic Intervention (N)	372	40	74	220	294	50
NL/WI	92/130	9/13	11/14	39/49	84/142	14/30
NL/WOI	135/242	15/27	30/60	77/171	83/152	8/20
RR(95% CI)	1.27 (1.08, 1.48)	1.25 (0.76, 2.04)	1.57 (1.08, 2.28)	1.77 (1.42, 2.20)	1.08 (0.89, 1.32)	1.17 (0.60, 2.26)
P-value	0.005	0.503	0.074	0.000	0.480	0.773
Uterine Contraction (N)	132	11	14	49	142	30
NL/WC	38/47	4/4	6/8	15/18	33/42	1/3
NL/WOC	55/85	5/9	5/6	24/31	51/100	13/27
RR(95% CI)	1.25 (1.01, 1.54)	1.80 (1.00, 3.23)	0.90 (0.53, 1.54)	1.08 (0.81, 1.43)	1.54 (1.20, 1.98)	0.69 (0.13, 3.60)
P-value	0.072	0.228	1.000	0.726	0.003	1.000

NL, Natural Labor; WI, With Intervention; WOI, Without Intervention; WC, With contraction; WOC, Without contraction; CI: Confidence interval

Regarding comparison of the mode of delivery, the results showed that the rate at which natural labor switched to cesarean section delivery at 40–40 + 6 and ≥ 41 gestational weeks was higher than that at 39–39 + 6 weeks. However the differences were not statistically significant (*p* = 0.059 and 0.071, respectively).

The natural labor rate was calculated as the total number of vaginal deliveries /total number of births and the P values were calculated for the difference of with/without intervention or with/without contraction in each gestational group. In patients without spontaneous uterine contractions, iatrogenic intervention significantly decreased the rate of cesarean section at 38–38 + 6 and 39–39 + 6 weeks (*p* < 0.001 and 0.005, respectively), but there was no difference in the 40–40 + 6 and ≥ 41 gestational weeks groups. In patients with spontaneous uterine contractions, the cesarean section rate was decreased significantly at 40–40 + 6 weeks (*p* = 0.003) than in the 39–39 + 6 gestational weeks group, but there was no significant change with other delivery times.

## Discussion

The present study analyzed data in patients with diet-controlled GDM. The rate of cesarean section was increased significantly at ≥ 41 gestational weeks, but it showed no difference between the 39–39 + 6 and 40–40 + 6 gestational weeks groups. The rate of natural labor switching to cesarean section was slightly increased in the 40–40 + 6 and ≥ 41 gestational weeks groups compared to that in the 39–39 + 6 group; the difference was not significant (*p* = 0.059 and 0.071 respectively). For the patients without spontaneous uterine contractions, the rate of vaginal delivery with iatrogenic intervention was increased significantly at 39–39 + 6 weeks (*p* = 0.005), but it did not increase at 40–40 + 6 and 41 weeks (*p* = 0.480 and 0.773 respectively). Finally, in the patients with spontaneous uterine contractions, the rate of vaginal delivery was increased significantly at 40–40 + 6 weeks (*p* = 0.003), and slightly increased at 39–39 + 6 weeks (*p* = 0.072), but the difference was not significant.

Deciding on the optimal timing of delivery requires balancing the conflict between maternal and fetal benefits and the risk of complications [20]. Joep et al. [21] found that the rate of adverse maternal–fetal risks was increased significantly after 41 gestational weeks for singleton pregnancies without complications such as hypertension or diabetes. Our study found that the cesarean section rate was significantly increased at 41 gestational weeks compared to that at 39–39 + 6 weeks. The rate of multiple adverse outcomes (macrosomia, III-polluted amniotic fluid, neonatal hypoglycemia) was slightly increased at 41 weeks compared to 39–39 + 6 weeks, although the difference was not statistically significant. Therefore, patients with diet-controlled GDM should not deliver after 41 weeks of gestation, which is also recommended for other singleton pregnancies.

As mentioned earlier, the recommended time of delivery for patients with medication-treated GDM is 39–39 + 6 weeks of gestation. This raises the question of whether delivery should also be indicated before 39 gestational weeks for diet-controlled GDM. In 2013, ACOG, gynecologists, and the Society for Maternal–Fetal Medicine [22] stated that they had long discouraged non-indicated delivery before 39 weeks of gestation, and diet-controlled GDM was not an indication. Several studies [11, 23, 24] found that the rate of access to neonatal intensive care units, hypoglycemia, weight loss, and cesarean delivery due to induction of labor were increased when delivery occurred before 39 weeks of gestation for diet-controlled GDM. In our study, however, there were no significant increases in adverse maternal and neonatal outcomes before 39 weeks of gestation when compared to those after 39 weeks, even the natural labor switching to cesarean section rate was slightly decreased in the 38–38 + 6 gestational weeks (p = 0.056). But with the absence of risks after 39 weeks of GDM, we do not recommend iatrogenic intervention before 39 weeks of gestation to avoid an early-term birth.

According to the analysis, the optimal delivery time for patients with diet-controlled GDM should be between 39–40 + 6 weeks of gestation. We analyzed the data of patients who underwent delivery during this period separately according to whether they had spontaneous uterine contractions. In patients without contractions, we found that the rate of vaginal delivery was increased significantly at 39–39 + 6 gestational weeks when given iatrogenic intervention, but there were no differences in the vaginal delivery rate at 40–40 + 6 gestational weeks. This is the same with a study involving Chinese pregnant women on induction of labor with dinoprostone in a patient with GDM [25]. Therefore, we recommend that iatrogenic intervention be performed at 39–39 + 6 weeks of gestation to induce labor. In patients with spontaneous uterine contractions starting at 40–40 + 6 weeks of gestation, the rate of vaginal delivery was significantly higher than that of cesarean section delivery. Otherwise, the rate of cesarean delivery was increased significantly at 40–40 + 6 weeks of gestation in the absence of spontaneous uterine contractions.

## Conclusions

Generally, we recommend the optimal delivery time for patients with diet-controlled GDM to be between 39 and 40 + 6 weeks of gestation. The Bishop score of patients should be assessed at 39–39 + 6 weeks of gestation, and for those with a Bishop score below 4, iatrogenic intervention is not recommended. Waiting for spontaneous uterine contractions under close management may be a better choice.

This study had some limitations. Although the data was collected from one of the largest hospitals in the region, this was only a single-center study. Patients at higher-rated hospitals usually receive better management than those at lower-rated hospitals, which may have led to bias in maternal and neonatal outcomes. Prospective and multicenter cohort studies may reduce bias and provide more convincing conclusions in the future.

## Data Availability

The datasets generated and/or analyzed during the current study are not publicly available due the private information of the patients but are available from the corresponding author on reasonable request.

## Abbreviations

GDM: Gestational diabetes mellitus
PROM: Premature rupture of membrane
ACOG: American College of Obstetricians and Gynecologists
SOGC: Society of Obstetricians and Gynecologists of Canada
OGTT: 75-Gram oral glucose tolerance test
FPG: Fasting plasma glucose
PPH: Postpartum hemorrhage
